# Emergence Delirium and Its Association with Preoperative Anxiety in Paediatric Patients Undergoing Infra Umbilical Surgery Under Combined General and Caudal Anaesthesia: An Observational Study from a Tertiary Care Centre in a South Asian Country

**DOI:** 10.5152/TJAR.2021.1104

**Published:** 2022-04-01

**Authors:** Aly Bahadur Ali

**Affiliations:** Department of Anaesthesiology, Aga Khan University, Karachi, Pakistan

**Keywords:** Caudal block, emergence delirium, modified Yale preoperative anxiety scale, paediatric anaesthesia emergence delirium, preoperative anxiety

## Abstract

**Objective:**

The objective of this observational study was to determine the incidence of emergence delirium (ED) and its association with preoperative anxiety using the modified Yale preoperative anxiety scale and paediatric anaesthesia emergence delirium scale, in a tertiary care institution in South Asia.

**Methods:**

A total of 250 children of 2–8 years of age (of either gender, American Society of Anaesthesiology classification I or II, undergoing infra umbilical surgery, using caudal block for analgesia) were enrolled and the study was completed. The primary outcome measure was the presence of ED using the paediatric anaesthesia emergence delirium score. A cut-off value equal to or more than 12 at any time point was taken as ED. The secondary measure was preoperative anxiety, measured using the modified Yale preoperative anxiety scale at 30 minutes before going into the operating room. A cut-off score of 30 was used.

**Results:**

The median age of the children was thirty-six months IQR (24–60). There were 230 (91%) males and 20 (9%) females. Twenty-two percent of the children experienced emergence delirium. One-point increase of modified Yale preoperative anxiety scale anxiety score was significantly associated with 1.23 times the odds of emergence delirium OR =1.23 (1.16–1.29) as compared with those without emergence delirium.

**Conclusions:**

The incidence of emergence delirium in our cohort was 22.4%, and there was a significant association between preoperative anxiety and emergence delirium.

**Main Points**

- Previous studies have suggested that there may be an association between preoperative anxiety and emergence delirium, but most of these studies have been done in the Caucasian population in high-income countries.
- There are very few studies from LMIC countries using validated tools on this topic. This study will add to the data from South Asia.
- In addition, cultural factors may also affect the incidence of postoperative delirium, and incidence may be different in different cultures. This point has been highlighted in this study.

## Introduction

Emergence delirium (ED) is a transient state of marked irritation and disassociation after awakening from anaesthesia exhibited by some patients.^1^ Stress and higher levels of anxiety are associated with a higher incidence of ED.^2^ It is also associated with other adverse outcomes like the increased intensity of postoperative pain, delayed recovery, and postsurgical disturbances in sleep-wake cycle.^2,3^

Cultural differences in parental behavior toward children undergoing surgery may have an effect on children’s anxiety levels and their postoperative behavior, which may account for differences in the incidence of ED in different cultures. To our knowledge, scant literature exists about the incidence of ED and its association with preoperative anxiety in Asian populations. Only a handful of studies have been conducted in Asian children, but not all authors have used validated scales for measuring anxiety.^4^

The primary objective of our study was to find the incidence of ED in the paediatric population undergoing infra umbilical surgery under combined general anaesthesia (GA) and caudal block using a validated paediatric anaesthesia emergence delirium (PAED) scale in a tertiary care teaching hospital in a South Asian country.^5^ The secondary objective was to see if an association existed between preoperative anxiety measured by using the modified Yale preoperative anxiety scale (mYPAS) and emergence delirium among these children.

## Methods

Ethical approval for this study (ERC No: 4986-Ane-ERC-17) was provided by the Ethical Review Committee of the Aga Khan University Hospital, Karachi, Pakistan (Chairperson, Professor Shaista Khan), on October 6, 2017.

The study was conducted between December 2017 and October 2018 in the preoperative holding area, operating room, and post anaesthesia care unit (PACU). Written informed consent was obtained from parents. Two hundred and fifty children aged between 2 and 8 years of either gender, American Society of Anaesthesiology (ASA) I or II undergoing elective infra umbilical surgeries, under combined GA and caudal block, were enrolled in the study. Children with developmental delay, neurological disease, those who had undergone previous surgery, and those who received premedication were excluded. There were no dropouts, and all children completed the study.

Regarding the sample size, the expected incidence of ED was taken as 20% from a previous study.^6^ To estimate the incidence with a precision of plus or minus 5%, 95% CI (15–25), 250 children undergoing elective infra umbilical surgeries were required.

In the preoperative holding area, the fasting period was recorded and the anxiety level of the children was evaluated using the mYPAS at 30 minutes before going into the operating room and in the presence of parents by either of the authors. A cut-off score of thirty was used to determine the presence of high level of anxiety.^7^ One parent was allowed to accompany the child to the operating room and was present at the time of induction.

Induction was standardized. After the application of ECG and pulse oximeter (Care Scape B650, Finland), induction was started with 8% sevoflurane in oxygen. Age and weight-appropriate laryngeal mask airway was inserted and anaesthesia was maintained using sevoflurane with 60% nitrous oxide and oxygen on spontaneous ventilation. Caudal block was administered in the lateral position using ropivacaine 0.25% 1 mL kg^-1^ (maximum cut-off dose was 3 mg kg^-1^). Total volume was limited to 20 ml. The efficacy of the caudal block was checked by the patient’s requirement for any further analgesia after the start of surgery.

Anaesthesia was maintained with sevoflurane and a Minimum Alveolar Concentration (MAC) value of 1.0-1.3, with 60% nitrous oxide and oxygen. Need for any further intravenous analgesics after the surgical incision was determined by a persistent increase in heart rate by more than 30% for 1 minute. Further analgesia (tramadol 1 mg kg^-1^ bolus) was administered as a rescue analgesic, if required.

At the time of application of wound dressing, laryngeal mask airway was removed after increasing the depth of anaesthesia with sevoflurane. After ensuring respiratory and hemodynamic stability, child was then shifted to PACU.

The children were evaluated in PACU for ED based on PAED scale^8,9^ by a trained research assistant. The scores were recorded from the time children were shifted to the PACU until the time he/she was shifted out, that was the time when the study ended. If the child was asleep “child sleeping” was mentioned in the scale column. Time of awakening (response to first verbal command) was also recorded and scoring was done at 15-minutes intervals till the discharge of the patient from the PACU. A cut-off of more than or equal to 12 points was used to determine the presence of ED.^10^ All children stayed in PACU for a minimum period of 60 minutes. Decision of when to treat ED was left to the discretion of the attending anaesthesiologist as it is considered a self-limiting condition but, if required, it was standardized to tramadol 1 mg kg^-1^.

Complications such as accidental removal of intravenous (IV) cannula or dressing and increased bleeding from the surgical site were also noted.

Data were analyzed by Statistical Packages for Social Science version 19 (SPSS Inc.; Chicago, IL, USA). Numeric point estimation was reported in mean and standard deviation for normality distributed data tested by Kolmogorov-Smirnov test and non-normal data were reported in terms of median with percentiles. Independent sample t-test or Mann Whitney U-test was applied for significant testing as per the distribution of data. Frequency and percentage were computed for qualitative observation and analyzed by chi-square or Fisher exact test. Logistic regression was performed and an odds ratio with 95% CI was computed. Multicollinearity was observed by using Pearson or Spearman correlation and *r* > 0.7 was considered multicollinearity. *P* ≤ .05 threshold was considered as significant.

## Results

A total of 250 children undergoing elective infra umbilical surgery were enrolled in the study. The median age of the children was 36 months, IQR 24–60. There were 230 (91%) males and 20 (9%) females. Moderate anemia (n = 21 [8.4%]) and asthma (n = 4 [1.6%]) were observed as comorbidity. Inguinal hernia repair and circumcision were the most common surgeries performed. Fifty-five children were diagnosed to have ED (22%). There was no statistically significant difference in demographic characteristics and type of surgery in patients with ED and without ED (Table 1 for demographic and other details). No difference was observed for liquid or solid fasting time between the two groups (fasting hours for liquid 5.09 ± 1.68 vs 5.36 ± 1.77; *P*  = .323 and for solids 8.31 ± 1.23 vs 8.38 ± 1.45; *P*  = .10). Additional intraoperative analgesia was required in three patients in the ED and for 10 patients in the non-ED group (*P*  = .35).

A high level of preoperative anxiety was present in 60 patients (24%), and the mean mYPAS score of these children was significantly high in the patients with ED (difference = 29.06), 95% CI 26.7–31.3 (Table 2). The univariate analysis showed a strong relationship between the mYPAS anxiety score and ED. One-point increase of mYPAS anxiety score was significantly associated with 1.22 times the odds of emergence delirium OR 1.22 (1.16 – 1.28) as compared with those without ED.

In multivariate analysis, a change was observed in odds ratio after controlling the effect of age, weight, gender, and surgery more than one. One-point increase of mYPAS anxiety score was also significantly associated with 1.23 times the odds of ED, OR = 1.23 (1.16 – 1.29) as compared to those without ED (Table 3).

Regarding complications, accidental removal of IV cannula did not occur in any patient. One patient in the ED group removed his dressing. Increased oozing from the surgical site occurred in four patients in the ED and none in the non-ED group (7.3% vs 0%; *P*  = .002) (Table 4).

## Discussion

The relationship between preoperative anxieties on ED is still controversial. Some studies have reported a positive correlation between preoperative anxiety and ED^2,11^ whereas others have not.^6^ The incidence of ED in our paediatric population undergoing infra umbilical surgery with caudal block was found to be 22.4%. There was no correlation of age, gender, and ASA status with the incidence of ED. Mean mYPAS of the children with ED was significantly high as compared to those without ED. There was a significant association between preoperative anxiety and ED. This study indicates that every two out of ten children experienced ED that could potentially lead to further complications.

Several factors like age, pain, gender, and parenteral presence can affect the incidence of ED. Studies have demonstrated that the younger the age of the child, the greater is the incidence of ED. The reported incidence in children aged between 2 and 6 years is up to 50%.^12,13^ The reason could be that the younger age group children are psychologically immature and therefore unable to cope with sudden awakening in an alien environment. However, we did not find any significant correlation of younger age with increased incidence of ED on multiregression analysis. This may be because in our institution the parents are routinely called to the premises of PACU once the child is wheeled in there.

Children with higher pain scores have also been shown to have a higher incidence of ED.^6^ We tried to standardize this and kept it constant by choosing surgeries performed under caudal analgesia and monitoring the failure rate.

Our study had more male patients (92%) as compared to female patients (8%). The reason was that majority of our patients underwent circumcision and inguinal hernia; both surgeries are more common among males. The percentage distribution of both these surgeries was equal in the group that had ED versus those who did not. Our analysis did not show any correlation of gender with the incidence of ED. In adult surgical patients, Munk et al.^14^ have reported male gender to have a positive correlation to ED, but the specific effect of gender on ED has not been adequately researched in paediatric patients.^[Bibr b14-tjar-50-2-129]^

Parental presence has a role not only in reducing preoperative anxiety^15^ at the time of induction but also in a subsequent reduction in ED;^16^ therefore, we kept this variable constant in our methodology and allowed parents to be present at the time of induction and in the PACU.

Much work has been done on ED in children from United States, Australia, and Europe.^17-19^ Driscoll et al.^17^ from United States reported an incidence of 43% with sevoflurane and 34% with desflurane anaesthesia in children undergoing otolaryngology procedures.^17^ Costi et al.^18^ from Australia reported an incidence of 29% following sevoflurane anaesthesia in children undergoing MRI scan.^[Bibr b18-tjar-50-2-129]^ Both studies used a cut-off score of ≥12 on PAED scale to detect ED. Doerrfuss et al.^19^ from Germany reported an incidence of 10.5% in children undergoing surgery with propofol, sevoflurane, or desflurane anaesthesia. They used midazolam as premedication and a cut-off score of ≥10 to detect ED.^[Bibr b19-tjar-50-2-129]^

In comparison very few studies on this topic have been conducted in the Asian population. Children in Asian cultures can be emotionally more dependent on their parents and hence may show different anxiety levels which may then have a potential effect on ED.

In our literature search, we were only able to locate one other study from South Asian region conducted by Sethi and colleague from India.^6^ We were unable to locate any prior study from Pakistan, or from any other South Asian country besides India. Sethi et al.^6^ compared the incidence of ED under sevoflurane and desflurane anaesthesia in children undergoing cataract surgery.^[Bibr b6-tjar-50-2-129]^ They used a cut-off score similar to ours, that is, ≥12 on PAED scale and reported an incidence of 20.45% and 18.18% with sevoflurane and desflurane anaesthesia, respectively.^6^ Our results are similar to Sethi’s, probably due to cultural similarity. However, a difference observed in Sethi’s study^6^ was that they observed no correlation between preoperative anxiety and ED in their population.^[Bibr b6-tjar-50-2-129]^ The incidence of ED in our study and that from India^6^ appears to be generally less than seen in those studies reported from United States, Australia, and Europe^17-19^ but a direct comparison is difficult due to methodological differences.

Other studies on ED from Asia have been reported from Singapore,^20^ Iran,^21^ and China.^22^ The incidence reported in these studies varies from 10% in Singapore,^20^ 18% in Iran,^21^ and 46% in the study done by Chinese authors.^22^

First, the strengths of our study are that we have used a validated scale.^5^ Studies using PAED scale, which is a validated scale,^5^ have reported a lower incidence of ED.^6,21^ Studies that have not used validated scales have reported a higher incidence of ED.^23^ Second, we used a more sensitive score of ≥12 to determine the presence of ED. A cut-off score of ≥12 has been shown to have greater sensitivity and specificity to detect ED.^10^

We also standardized several of the factors that may affect ED, for example, parental presence, type of surgery, and pain. In addition, very few studies have been reported from South Asia, and this study will add to the paucity of this literature.

Our study has some limitations, foremost that ours was an observational study and suffers from the disadvantages of this type of study. We measured PAED scores at 15-minute intervals. It may have been more precise to have recorded scores at shorter intervals with increased duration of follow-up. Costi et al.^18^ and Bong et al.^24^ have used an interval of 5 minutes for recording PAED scores in their studies. Another limitation of our study was that we did not standardize the gender of the parent accompanying the child to the operating room or postoperatively in the recovery room although this did not influence our results in the multilinear regression model. To the best of our knowledge, the effect of parental gender has not been studied so far. The use of BIS monitoring to standardize the intraoperative depth of anaesthesia could have improved the scientific validity of our study; however, this was not done due to budgetary constraints.

There is no consensus on treatment as ED is a self-limiting condition. We used the analgesic tramadol for additional analgesia if required because of its availability. Availability of fentanyl or morphine is an issue in many LMIC countries.

In conclusion, the results of our study indicate that the incidence of ED in our paediatric population aged 2–8 years undergoing infra umbilical surgery with a caudal block is 22.4%, and a significant association was observed between preoperative anxiety and ED. We recommend that the effect of cultural differences on ED be further investigated in future studies.

## Figures and Tables

**Table 1.** Demographic Data and Other Variables (n = 250)

Variables	ED, n = 55	No ED, n = 195	*P*
Age (months)	36 [24–60]	36 [24–60]	.551
Weight (kg)	15 [11–20]	15 [12–18]	.741
Height (cm)	98 [88–109]	98 [88–110]	.997
BMI (kg m−2)	15.12 [13.4–16.8]	15.5 [14–17]	.292
Gender			
Male	50 (90.9%)	180 (92.3%)	.736
Female	5 (9.1%)	15 (7.7%)
ASA			
I	41 (74.5%)	147 (75.4%)	.899
II	14 (25.5%)	48 (24.6%)
Procedure and time			
Single procedure	50 (90.9%)	178 (91.3%)	.931
Two procedures	5 (9.1%)	17 (8.7%)
Anaesth time (minutes)	63.91 ± 20.35	64.39 ± 17.61	.862
Surgical time (minutes)	41.55 ± 20.35	41.37 ± 16.25	.948

Data are presented as median, IQR (25–75), mean ± SD, and number of cases (%)

ED, emergence delirium; ASA, American Society of Anaesthesiology classification; Anaesth, anaesthesia; BMI, body mass index.

**Table 2.** Comparison of Outcome Between ED and Non-ED Patients (n = 250)

Variables	ED, n = 55	No ED, n = 195	Mean Difference, [95% CI]	*P*
mYPAS Scale	55.24 ± 12.18	26.18 ± 5.65	29.06 [26.77–31.34]	< .01^*^
PAED Scale	13.67 ± 1.53	7.52 ± 1.48	6.15 [5.7–6.59]	< .01^*^

ED, emergence delirium. ^*^Significant difference.

**Table 3.** Multivariable Logistic Regression Analysis to Predict Emergence Delirium (n = 250)

Predictors	Adjusted Odds Ratio	95% CI	*P*
mYPAS Scale	1.23	1.16–1.29	.0005^*^
Age (months)	0.36	1.01–1.04	.387
Weight (kg)	1.11	0.91–1.36	.290
Gender			
Male	0.559	0.07–4.21	.572
Female	Ref.
Surgery			
Single	Ref.	0.089–12.27	.972
Multiple	1.05

mYPAS, age and weight as continuous independent variables in the model. Nagelkerke R square = 0.788; Model accuracy = 95.6%. ^*^Significant difference. Ref., Reference for comparison

**Table 4.** Complications Seen Between ED and Non-ED Patients

Variables	ED, n = 55	No ED, n = 195	Difference [95% CI]	*P*
Complication				
Removal of IV cannula	0 (0%)	0 (0%)	NA	NA
Removal of dressing	1 (1.8%)	0 (0%)	1.8% [−1.7%–5.3%]	.220
Increased bleeding from surgical site	4 (7.3%)	0 (0%)	7.3% [0.4%–14.2%]	.002^*^

NA, not applicable; ED, emergence delirium. ^*^Significant difference.
